# Models of Protective Immunity against Schistosomes: Implications for Vaccine Development

**DOI:** 10.3390/pathogens12101215

**Published:** 2023-10-03

**Authors:** R Alan Wilson

**Affiliations:** 1 Department of Biology and Biomedical Research Institute, University of York, York YO10 5DD, UK; alan.wilson@york.ac.uk; 2 Programa de Pós Graduação em, Ciências Biológicas, Universidade Federal de Ouro Preto, Ouro Preto 35402-136 , Brazil

**Keywords:** *Schistosoma*, vaccine, mouse, rat, rhesus macaque, radiation attenuation, self-cure, peptide array, systems biology, adjuvant

## Abstract

After many decades of research, a schistosome vaccine still looks to be a distant prospect. These helminths can live in the human bloodstream for years, even decades, surrounded by and feeding on the components of the immune response they provoke. The original idea of a vaccine based on the killing of invading cercariae in the skin has proven to be illusory. There has also been a realisation that even if humans develop some protection against infection over a protracted period, it very likely involves IgE-mediated responses that cannot provide the basis for a vaccine. However, it has also become clear that both invasive migrating larvae and adult worms must expose proteins and release secretions into the host environment as part of their normal biological activities. The application of modern ‘omics approaches means that we now have a much better idea of the identity of these potential immune targets. This review looks at three animal models in which acquired immunity has been demonstrated and asks whether the mechanisms might inform our vaccine strategies to achieve protection in model hosts and humans. Eliciting responses, either humoral or cellular, that can persist for many months is a challenge. Arming of the lungs with effector T cells, as occurs in mice exposed to the radiation-attenuated cercarial vaccine, is one avenue. Generating IgG antibody titres that reach levels at which they can exert sustained immune pressure to cause worm elimination, as occurs in rhesus macaques, is another. The induction of memory cell populations that can detect trickle invasions of larval stages remains to be explored. One promising approach is the analysis of protective antibodies using high-density peptide arrays of target proteins to identify reactive regions. These can be combined in multi-epitope constructs to immunise a host against many targets simultaneously and cheaply.

## 1. Why Are Schistosome Vaccines So Problematic?

It is almost an axiom that if exposure to an infectious agent results in a rapid cure that induces strong long-lasting immunity against a second exposure, then an effective vaccine is a feasible proposition (think measles or smallpox). Conversely, if an infection follows a chronic time course with little evidence for protection against further exposure, then developing a vaccine will be an onerous task. Schistosomes surely fall into this second category. The problem is compounded by, and indeed possibly related to, the fact that helminths do not multiply in the human host. The elicited immune response depends on the frequency with which infective larvae are encountered and the size of the burden acquired. There is anecdotal evidence from travellers that adult *S. mansoni* can persist for years, even decades, in infected humans [1,2], while a study of *S. haematobium* in The Gambia estimated the mean worm lifespan as 3.4 years [3]. The existence of a protective immune response in humans was initially suggested by the shape of the age–intensity curve in endemic communities. This curve characteristically shows a rise in intensity during the first two decades of life, followed by a decline in adults to very low levels. Was this an immunity to reinfection, a reduction in exposure in older individuals, or both? [4]. Treatment and reinfection studies revealed that children rapidly became reinfected but adults did not, leading to the idea that protective immunity took years to develop. This hypothesis was counteracted by observations on naïve populations migrating into endemic areas. 

Very early in these human studies, a strong correlation between IgE response and a 22.6 kDa protein allergen was established in all three main human schistosome species (see [5]). The protein, which possesses two EF-hand calcium domains, was localised by immunocytochemistry to the tegument, gastrodermis, and nephridial canals [6]. Two similar proteins with Mw of 21.7 and 20.8 kDa were also identified. Although Sm22.6 is not confined to the tegument, these three proteins were later renamed as TAL (Tegument-Allergen-Like) constituents of a 13-member family in *S. mansoni* [5]. A similar family has recently been characterised in *S. haematobium* [7]. Inherent problems with Sm22.6, the principal allergen, are its situation within the tegument cytoplasm of adult worms and lack of expression in the penetrating cercaria or early schistosomulum. A consensus appears to be emerging about the potential role of IgE in protective immunity in humans. Stimulation of IgE by released allergens is posited to result from the death of adult worms due to senility; chemotherapy may hasten the process. In addition, a degree of cross-reactivity is required between adult and larval TALs to trigger an immediate hypersensitivity response to incoming larvae that prevents their establishment. How this works to block or kill larvae is unknown. As Oettle et al. acknowledged [7], antigens like Sm22.6 that stimulate IgE production cannot be considered as vaccine candidates because of the association between IgE and anaphylactic responses. The demise of the promising Na-ASP-2 hookworm vaccine vividly illustrates the problems of working with potential helminth allergens [8]. 

Preventing parasite establishment at the point of entry is a very attractive proposition, but there is an almost total lack of evidence for schistomula death in the skin in any naïve or immune host species. This issue was reviewed by Wilson, Li, and Castro-Borges [9]. The concept gained enormous traction from the development of an in vitro antibody-dependent cellular cytotoxicity (ADCC) assay [10]. Artificially transformed 3hr old schistosomula are highly susceptible to killing by a combination of antibody isotypes and effector leukocytes in vitro (127 papers in PubMed in the 26 years between 1974 and 1999). However, it appears that invading larvae avoid the ADCC trap in vivo. They remain distal in the epidermis after skin entry [11] while, of necessity, exposing internal proteins when shedding cercarial membranes during transformation to schistosomula [12]. This brief window of susceptibility closes in <24 h as the parasite acquires its new non-reactive surface [13]. Indeed, remaining peripheral in the skin for >40 h [14] beyond the reach of antibodies and leukocytes can be considered an immune evasion strategy. A single histopathological study of the skin-invasion phase in the olive baboon [15] provided visual evidence for dead schistosomula in the epidermis of animals with long-term *S. mansoni* infection. However, many unaffected larvae that failed to attract an inflammatory focus were also present. The authors concluded that the immune mechanism took months to develop and its impact on worm burden may have been minimal since infected baboons showed little protection against challenge, ranging from 17 to 22% [16,17,18]. The advent of parasite-tracking techniques revealed that worm elimination occurred after the skin phase in both naïve and immune mice, [19] but these experiments failed to stop the ADCC juggernaut. Arguably, this gulf between in vitro studies and in vivo reality stifled the development of schistosome vaccines for decades, and its influence lingers to the present day. If a rational basis for schistosome vaccine development is desired, then, in absence of a useful paradigm based on human responses, we need to focus on laboratory models in which acquired immunity can be demonstrated. This review asks which features of the irradiated cercarial vaccine in rodents and primates, self-cure in the rat (*Rattus norvegicus*), and self-cure in the rhesus macaque (*Macaca mulatta*) underlie the development of protective immunity.

## 2. The Radiation-Attenuated (RA) Vaccine Model

Summaries of research on the model can be found in detailed reviews by James [20], Coulson [21], Hewitson et al. [22], and Bickle [23]. Most information about the dynamics and mechanism of the model has come from mouse experiments. Cercariae are attenuated by various forms of ionising radiation (gamma rays, X-rays), while exposure to radiation over a short period is crucial. With too little radiation, attenuated parasites reach and develop in the portal system, leading to misinterpretation of results [24]. With too much, they are confined to the skin, failing to elicit protection [25]. A crucial feature of the model is that it allows a large dose of larvae, which would normally be fatal, to be applied (typically 500 for a mouse, 1000 for a rat, and 9000 for a primate like the baboon). In our studies with gamma rays from a Co^60^ or X-ray source, 200 Gy (20 krad) was optimal. This allowed vaccinating parasites to exit the mouse skin, some via draining lymph nodes, and reach the lungs, where they persisted and recruited CD4+ memory effector cells to arm that organ [26]. Detailed information is not available for primate hosts, which must be treated as a “black box” in terms of mechanism.

Unfortunately, the laboratory mouse has intrinsic flaws as a vaccine test bed [27]. Briefly, the arguments are as follows. The low level of maturation of penetrating cercariae (~32% for *S. mansoni*) is a major limitation of the model since 68/100 parasites fail to mature in naïve mice due to natural causes. The pulmonary capillary bed presents a particular obstacle to intravascular migration en route to the portal system. The fragility of pulmonary capillaries and their susceptibility to cytokine-induced vascular leak syndrome result in schistosomula bursting into the alveoli from which they possess only limited capacity to re-enter tissues. A single exposure of attenuated schistosomula arms the lungs by recruiting persistent memory/effector CD4+ Th cells to the pulmonary parenchyma. The interval between the last vaccination and challenge has conventionally been set at 5 weeks, precisely to allow inflammatory processes to subside before the primed mouse encounters a normal challenge. The arrival of a normal challenge parasite in the lungs rapidly triggers a cellular effector focus, which blocks its onward migration, increasing the probability of deflection into an alveolus. It must be emphasised that the trapping of migrating schistosomula in the lungs, associated with inflammatory foci, is not in itself a lethal killing event. An ultrastructural study of such challenge schistosomula found minimal evidence for cytological damage [28]. Moreover, trapped lung schistosomula, which would not mature if left in situ, could do so if transferred to the vascular system of naïve mice, confirming their viability [29]. Two studies indicated that this pulmonary phase immunity in mice, induced by a single exposure to attenuated cercariae, was reasonably persistent. In the first, challenge at 15 weeks resulted in no reduction in 78–80% protection [30]. In the second, protection was around two-thirds of the original level at six months but had declined to zero by one year [31].

A single exposure of mice to the RA vaccine elicits a predominant Th1 response, with subsequent exposures augmenting Th2 involvement and the contribution of the antibody as an effector. It has proven to be difficult to recreate pulmonary cellular responses using either adoptive transfer of cells or vaccination with antigen preparations that would populate the lungs with effector cells. Multiple vaccinations of C57Bl/6 mice were needed to raise antibody titres to the level at which immunity could be passively transferred to naïve recipients (as well as massive amounts of serum equating to several vascular volumes delivered iv and ip) [32]. This study emphasised the importance, even dominance, of the cellular component. The Th1:Th2 balance elicited by the RA vaccine is probably the important determinant, as recently shown with 50% protection achieved by passive transfer of sera from IFNgR KO mouse donors with a highly skewed Th2 response. Protection in donor mice was correlated with antibody titres 2.5× those achieved in comparable C57Bl/6 donors [33]. 

The problem with most single antigen vaccine protocols in the laboratory mouse, compared to the attenuated vaccine, is the short interval, sometimes only 10 or 14 days between the last antigen boost and cercarial challenge of test and control animals. This means challenge parasites will reach the lungs when both activated T cells and cytokine levels resulting from the preceding vaccination are maximal in the circulation. It was suggested [27] that “protection” in this situation was the result of physiological effects on pulmonary blood vessels, increasing the proportion of parasites that enter the alveoli. This hypothesis explains why internal antigens, which are unlikely to interact with the immune response in living schistosomula, plus a variety of heterologous proteins, can reduce the level of maturation in a non-antigen-specific way.

In view of what follows in the section on rat self-cure, it is worth considering whether nitric oxide (NO) has a potential role in RA vaccine-induced parasite elimination before leaving the mouse model. Immunologically activated murine macrophages were shown to kill skin-stage schistosomula of *Schistosoma mansoni* in ADCC assays but not by surface adherence like granulocytes [20]. Rather, the cytotoxicity was directed against internal organelles, especially mitochondria [34]. Moreover, juvenile parasites recovered from the liver were also partially and transiently susceptible to activated macrophage-mediated cytotoxicity in vitro, while, in contrast, ex vivo lung schistosomula were completely refractory to killing in such assays at day 7 or 10 [35]. Subsequently, the active agent released by activated macrophages was demonstrated to be nitric oxide (NO), acting via enzymes required for aerobic energy metabolism, thus making the link to mitochondria [36]. Unfortunately, an intrinsic flaw of the in vitro assays was revealed when the addition of erythrocytes (at 1/200 dilution of whole blood) to larvicidal assays was found to abolish the cytotoxic killing of newly transformed schistosomula [37]. This paralleled the blocking effects of erythrocytes on the antiproliferative action of NO on another extracellular blood parasite, *Trypanosoma brucei* [38]. This should mean that once schistosome larvae enter the bloodstream, they are unlikely to be exposed to cytotoxic concentrations of NO, irrespective of their energy metabolism. The independent derivation of inducible nitric oxide synthase-deficient mice (iNOS KO) facilitated the function of NO in the RA vaccine model to be investigated directly in two studies [37,39]. One reported no significant reduction in protection induced in iNOS KO versus wild-type mice [37]. The other found that vaccine-induced protection was reduced by 25 to 30% [39]. The interpretation of results was complicated in both studies by the observation of higher worm burdens in both vaccinated and control iNOS KO mice compared to wild-type groups, possibly due to altered pulmonary vascular physiology. It was suggested that challenge schistosomula deflected into the alveoli would be not be protected by erythrocyte quenching of NO from macrophages by about 18 days post infection [39]. Conversely, by then it was too late, as they had entered the alveoli anyway and migration to the portal vein was virtually complete [37]. It is no longer disputed that elimination of challenge parasites in the mouse model occurs in the lungs, but as dead/damaged parasites have not been described in ultrastructural [28] or histopathological studies [40] of the lungs, blocked migration would appear to be an adequate explanation.

It should be possible to overcome these limitations of the RA vaccine in the mouse model using larger rodents or primates with more robust pulmonary capillaries. There have been several studies with laboratory rats that have been complicated by the self-cure process around 28 days post infection (see Section 3 below). Using a combination of labelled-parasite tracking and portal perfusion (so as not to miss any schistosomula), the percentage of infecting parasites that reached the portal system at day 22 was determined as 39 to 42% [41]. (It is necessary to assess acquired immunity in the rat early after challenge, before self-cure kicks in.) Nevertheless, optimally irradiated parasites were found to travel to the lungs of rats exposed to the RA vaccine, where they persisted for at least 14 days. Elimination of challenge parasites occurred there too between 7 and 10 days post challenge [42,43]. Furthermore, passive transfer experiments implicated the IgG2a antibody as the effector isotype, not IgG2c or IgE [44]. The striking feature of this lung-phase immunity elicited in rats by the RA vaccine was that it persisted for at least six months, whereas the immunity induced by self-cure (see Section 3 below) was no longer significant by this time [42]. The Syrian hamster showed a better level of cercarial maturation, with a mean 56% recorded in eight experiments [27] and values as high as 76% in single experiments, but it does not appear to have been explored as an RA vaccine recipient.

The most compelling evidence for the utility of the RA vaccine as a paradigm comes from its administration to primates, in which worm maturation is not a problem. A value of 82% penetrating cercariae reaching maturity was recorded in vervet monkeys (*Cercopithecus aethiops*) [45]) and a mean value of 80.5% was found in three baboon (*Papio anubis*) experiments [46]. At this level of maturation, <20 out of 100 parasites died of “natural causes” during migration. A single exposure of primate hosts to 9000 cercariae did not elicit protection (c.f. the mouse or rat). Multiple exposures (3–5) elicited strong antibody-mediated protective immunity [45,46], which could be as high as 86% after 5 vaccinations based on recovered worm burden [47]. In this situation, a further 69 out of 100 worms died due to the immune effector response, the converse of the mouse situation. There was no evidence for arming of the lungs by attenuated parasites, as occurs in the mouse and rat. This may simply be a matter of scale: a 5 kg baboon would need a dose of 1.25 × 10^5^ attenuated cercariae to match the 5 × 10^2^ dose that arms the lungs in a mouse. The antibody responses over the time course in baboons were informative, with early IgM production that declined towards the background with each vaccination. The schistosome-specific IgG response showed a sawtooth pattern of boost and decline with successive vaccinations, progressively rising to a maximum at three weeks after the last exposure. Challenge at this time of maximum titre resulted in a 73% reduction in worm burden, but when delayed to 12 weeks, the reduction was 53% [47]; notably, the specific IgG titre declined by 66% over this interval. 

These data illustrate a major problem for schistosome vaccines that, in general, is not apparent from most vaccine experiments. Once stimulation of the immune system by the vaccine is removed, antibody titres decline. (This is the same problem with most current COVID-19 vaccines, hence the need for multiple boosters.) Almost always, the challenge is timed to coincide with peak titre and no information is provided about the duration of protection. The other important factor is induction of memory, which needs to be triggered by incoming larvae. In the endemic situation, these larvae will arrive in a trickle, not as the massive boost provided by a large cercarial bolus exposure. The need for the immune system to be on permanent “red alert” to deal with incoming cercariae was previously highlighted [48]. This has always been a problem unacknowledged by proponents of killing in the skin. There is simply no time to trigger a recall response before the cercariae transform to schistosomula and acquire their protective disguise. The experiments with multiple-vaccinated baboons bear this out—a secondary antibody response to a bolus challenge is hard to detect until larvae have reached the blood-feeding stage and are well established.

Unlike the attenuated malaria sporozoite vaccine [49], the RA cercarial vaccine has not been taken forward to human trials. With a cercarial lifespan of only hours [50], it would need a massive production facility. Their multicellularity means that cryopreservation of viable larvae has been hard to achieve (See [51] for review). Most importantly, given the intravascular migration route, we have no idea of the radiation dose that would permit larvae to reach the lungs but travel no further. Judging from chimpanzee experiments, 3–5 × 10^4^ larvae would be required to achieve protection [52]. The prospect of tens, hundreds, perhaps even thousands of attenuated larvae lodging in the human brain and nervous system does not bear contemplation. The RA vaccine model is best left as a paradigm of protective immunity to be explored for insights.

One aspect of the RA vaccine model that has received recent attention is the identification of the glycan and protein targets of protective antibodies using microarrays printed on glass slides [33,53]. The glycan array comprises a library of naturally occurring schistosome-derived N-, O- and glycosphingolipid (GSL) glycans isolated from different life stages. Mature schistosome eggs are a potent source of highly antigenic glycans, but vaccination with viable schistosome eggs does not elicit protection in different animal models [54]. This observation led to the smokescreen hypothesis suggesting that antigenic glycans deflect immune responses away from critical and susceptible protein epitopes [55]. However, the presence of eggs does not compromise the efficacy of the RA cercaria vaccine in baboons [56]. Many larval glycan epitopes are shared with eggs, but perhaps anti-larval antibody responses differ in some respects from those induced by eggs. It is notable that high titres of IgG against multifucosylated glycan epitopes were elicited by the RA vaccine in the absence of eggs [53]. These glycan structures would be worth pursuing in baboon vaccine experiments.

The peptide array comprises overlapping 15 mer peptides covering the complete sequence of 55 tegument and alimentary tract proteins [33]. Target selection was based on extensive RNASeq and proteomic studies of larval and adult worms to identify secreted and surface-exposed proteins putatively accessible to immune effectors in live parasites. Unsurprisingly, sera from IFNgR KO mice proved to be more reactive with targets than those from C57Bl/6 counterparts exposed to the same triple vaccination regime. Proteins from the alimentary tract were more reactive than those from the tegument, quite possibly a reflection of the amounts of material released into the bloodstream from the two tissues. Pairs of peptides were selected from 44/55 of the arrayed proteins based on their reactivity score and accessibility, which was indicated by mapping to crystal structures where possible (26/44). This information is currently being used to design multi-epitope vaccine constructs for vaccine trials. Major reactivities have been included in the construct from the esophageal glands (MEGs 4.1, 4.2, 8.1, 8.2. 8.3, and 12), the gastrodermis (apoferritin, cathepsins S, B1, and B2; asparaginyl endopeptidase) and the tegument (Sm13, Sm25; and ADP-ribosyl cyclase).

A systems biology approach has been used to identify the immune system components and pathways that correlate with the induction of protective immunity by vaccination for a number of infectious agents (e.g., yellow fever and influenza [57,58,59]). Farias et al. [60] recently used this approach to investigate the impact of the RA vaccine in mice. They found that the upregulation of hemostasis pathways after vaccination may contribute to parasite blockade in the lungs. It was notable that single exposure to attenuated parasites revealed early establishment of a Th1 bias and prominent encoding chemokines and their receptors, indicating an enhanced capacity for inflammation, again potentially augmenting the inhibition of intravascular migration. Increasing the number of vaccinations from one to three did not proportionally elevate protection, and there was a clear shift towards antibody-mediated effectors while elements of the early Th1 bias remained. Notable features after three vaccinations were markers of cytotoxicity (including IL-6 and NK cells), together with growth factors and their receptors (FGFR/VEGF/EGF) and the apoptosis pathway. Indeed, there was evidence for the development of anergy after three vaccinations, reinforced by the limited responses detected in 3× vaccinated animals after challenge. It was inferred that persistence of a Th1 response puts a limit on the expression of antibody-mediated mechanisms. This tendency to anergy may explain the failure of multiple vaccine doses to drive protection towards sterile immunity. These observations hark back to the “happy valley” hypothesis of 1999 [61], fleshing it out with details of potential underlying mechanisms. The contention was that schistosomes thrive best in the intermediate zone between Th1 and Th2 extremes. The attenuated schistosomulum is a large and complex vaccine “capsule” that interacts with the host skin, draining lymph nodes, immune elements in the circulation such as PBMC, and the pulmonary vascular network. It is probably not simply a matter of identifying antigenic targets. Vaccine strategies that seek to replicate the mechanisms of the RA vaccine using antigenic constructs may need a subtle formulation. Our experiments suggest that it may be possible to favour one T helper extreme or the other, but activating both simultaneously may not be feasible; sterile immunity by either extreme seems a long way off, in this model.

## 3. Self-Cure in the Laboratory Rat (*Rattus norvegicus*)

Interest in self-cure by Fisher rats from *S. mansoni* infection was stimulated by the detailed study conducted by Phillips et al. in 1975 [62]. Worm numbers in the portal system rose to a peak at 28 days and then declined to very low levels by 6–8 weeks. Cure was not absolute, as a few worms remained even at 6 and 12 months (1% and 0.5% of applied cercariae, respectively). RNASeq analysis of gene expression in such survivors would be informative about a worm’s ability to resist immune attack. This study [62] was performed not long after the basis of immediate hypersensitivity (allergy) was established by the identification of IgE class immunoglobulins and their ability to sensitise mast cells [63]. It is important to remember that IgE is essentially a tissue antibody produced locally by plasma cells to bind mast cells and basophils via their Fc epsilon receptors. It is present in the circulation at high levels after infection with intestinal nematodes like *Nippostrongylus*, and its association with expulsion of the parasite load was established [64], including degranulation of mucosal mast cells and increased permeability of the gut wall. A high level of total IgE production in helminth-infected rats, due to potentiation of a previous sensitisation to different non-parasite antigens, was also documented [65]. This potentiation of IgE responses to bystander antigens is a powerful argument against potential allergens as components of worm vaccines.

Inevitably, the production of IgE by schistosome-infected rats was also reported [66]. The profile of total IgE rose to a maximum at 42 days and remained high long after most worms had been eliminated. The habitat of schistosomes is not like that of *Nippostrongylus* in the gut lumen attached to the mucosal surface. Prior to maturity and mating, worms are located in the hepatic portal distributaries of the liver. If IgE is the specific mediator of worm killing, how does it operate? It was established that pronounced hepatic mastocytosis occurred in the rat, concomitant with parasite elimination [67]. The majority of recruited hepatic mast cells contained rat mast cell protease II, a useful indicator of mast cell degranulation, which was released into the bloodstream during the period of parasite elimination. This was quite different to the situation in schistosome-infected mice in which intraepithelial mastocytosis in the gut wall occurred later, in response to egg deposition. Furthermore, these intraepithelial cells were typical mucosal mast cells containing mast cell protease I. 

Mating of males and females should occur around 35 days, but elimination of worms in the rat begins at 28 days, before they begin to travel up the portal vein, taking themselves out of the hepatic danger zone. The total IgE level detected in rat circulation at 42 days after exposure to 2000 cercariae was equivalent to that in rats exposed to *Nippostrongylus* [68]. A rat basophilic cell line was sensitised with rat infection serum in vitro to measure the release of labelled serotonin after the addition of schistosome antigen. This confirmed the ability of schistosome protein to trigger serotonin release from IgE-sensitised cells at days 28 and 35. It was also observed that mast cells were degranulating in vivo by measuring the release of mast cell protease II into the bloodstream, with high levels at days 28 and 35 that subsided by day 42. 

Western blotting with immunopurified rat IgE against worm homogenate revealed two dominant allergens of 67 and 36–38 kDa in the mixture [68]. Periodate treatment with antigen preparations strongly inhibited mast cell degranulation in vitro, suggesting that the relevant antigenic epitopes were glycans. This is perhaps not surprising since degranulation requires the crosslinking of Fc epsilon receptors, which could be accomplished by repeating glycan structures. From these results, it was inferred that IgE-mediated effector responses were involved, at least in part, in the self-cure of rats. How mast cell products might kill adult worms was unclear. They could inflict direct damage (e.g., mast cell proteases), but the potential effects of biogenic amines (histamine and serotonin) are more intriguing. *S. mansoni* possesses a putative histamine receptor [69] and several putative serotonin receptors (https://www.uniprot.org (accessed on 1 September 2023)). These products could mediate hyperstimulation potentially via the nervous system, leading to exhaustion and death [48]. 

Collectively, it was difficult to see how these numerous observations on self-cure in the rat could inform schistosome vaccine development—the involvement of IgE, allergens, and mast cells was not promising. However, after a gap of 20 years, a completely new angle on *S. japonicum* worm expulsion was provided by experiments with inducible nitric oxide (NO) synthase knockout (iNOS−/−) rats [70]. Following infection, iNOS−/− rats showed 4-fold greater worm burden and 22-fold greater egg deposition in the liver compared to wild-type rats. Female fecundity was 5-fold higher, and worms recovered at seven weeks were significantly larger than those from wild-type rats. The reproductive organs of both male and female schistosomes from iNOS−/− rats had a more normal appearance and their uteri were filled with eggs. The authors concluded that in wild-type rats, nitric oxide synthesised by iNOS inhibited parasite growth, reproductive organ development, egg production, and viability, achieved by interfering with mitochondrial function. Later experiments suggested that the situation was more complicated [71]. There was a significant decrease in *S. japonicum-*elicited Th2/Th1 responses and cytokine and chemokine-producing capability in infected iNOS−/− rats. Is it just an accident of evolution that *Rattus norvegicus* has high levels of endogenous nitric oxide that impose immune pressure on pre-adult worms, resulting in their death? Was the hepatic mastocytosis described in Fisher rats [67] absent in iNOS−/− Sprague–Dawley rats? Is there a message here about the mechanism of adult worm elimination in rhesus macaques?

## 4. Self-Cure in the Rhesus Macaque (Macaca Mulatta)

The features of the model were established in the 1960s in experiments using small numbers of animals, so they were not amenable to statistical analysis (e.g., [72,73]). Briefly, primary exposure to between 100 and 1600 cercariae resulted in patent infection, judged by faecal egg excretion from five weeks. However, starting around 10 weeks, egg output declined to very low levels. Following challenge with 2000 cercariae at 21 weeks, all animals were completely protected compared to a control. A lower primary dose of 25 to 100 cercariae did not adequately prime the animals, but both primary and challenge parasites were eventually eliminated. Smithers and Terry [73] believed that the persistence of primary worms was necessary for the maintenance of immunity to challenge and coined the term “concomitant immunity,” although it is unclear whether they ever perfused their animals to find out. However, worm transfer experiments inferred the presence of host molecules on the adult worm surface as potentially being an immune evasion mechanism. From recent proteomic studies, we now have a much clearer idea of the identity of these host molecules (e.g., CD44 [74]). 

After an interval of 40 years, we revisited the rhesus macaque model using both *S. mansoni* [75] and *S. japonicum* [76] infections in groups of six animals, with strikingly similar outcomes between the two schistosome species. Large numbers of adult worms were established after exposure to 1000 cercariae, with maturation estimated at 44–65% of penetrants in macaques perfused at 8 weeks in an independent experiment [77]. Oviposition began at week 5, but from ∼8–10 weeks, egg excretion in faeces tailed off. We were able to estimate worm burden by assaying a circulating antigen (CAA) released from the worm gut as a surrogate. A decline in circulatory CAA levels indicated a cessation of blood feeding. At the 18-week perfusion time, the six macaques exposed to *S. mansoni* fell into three distinct groups with low, medium, and high worm burdens, which were inversely correlated with antibody titres. Strikingly, many recovered worms were pallid and emaciated; they appeared to be starving to death. In the subsequent experiment with *S. japonicum*, the six macaques could not be stratified on the basis of worm burden at 22 weeks or on antibody titre. However, we were able to document the severe size reduction of worms, especially females, compared to worms from mice, with shrinkage of ovary and testes, and fewer eggs in the uterus and sperm in the seminal vesicle. These morphological changes in both schistosome species were attributed to immunological pressure, with the conclusion that it took several weeks for worms to die. This was clearly not the same as the rapid worm death in rats that occurred over a period of days. Adult worms acquire nutrients across both their surface and via the gut, the latter being more important in females with their greater needs owing to egg production [78]. The female’s response to cessation of blood feeding appears to be the gradual resorption of non-essential internal organs, leading to shrinkage of the body, while the shrinkage of males is less marked [75]. Ultimately, both sexes succumb to the pressure.

A more comprehensive study of *S. mansoni* primary infection and subsequent challenge was recently published, using a cohort of 12 animals in a standardised protocol with modern immunological and parasitological techniques [79]. The first worms were estimated to arrive in the portal system on day 6; blood feeding began on day 8; the first females began egg laying 26 days later on day 34, with egg excretion at day 42; and the last arrivals matured around day 54. Based on the peak CAA level in each animal, worm maturation was estimated to be between 30 and 70% of penetrating cercariae. There was wide variation between animals, with the fastest responders affecting female fecundity by week 8. The pivot point at week 10 was followed by an exponential decline in egg excretion at approximately twice the rate of CAA levels. This meant that worms spent some time in a non-fecund state before expiring. The time taken after week 10 for half of the worms to die was 2.8 weeks in the fastest responder and 8.7 weeks in the slowest. This was an important point since it indicated that the process of elimination was very protracted. Schistosomula exposed to rhesus plasma before analysis of epigenetic gene expression revealed that the poorly understood regulation of autophagy may be an important target in immune pressure. It may be pertinent that autophagy and tissue remodelling occur at three points during early larval development, including resorption of the spent acetabular glands in the skin, body elongation in the lungs, and switch to blood-feeding juveniles in the liver. Interference in these processes by immune pressure could retard migration and development. 

Following challenge at 42 weeks, near background CAA levels in fast responder animals provided evidence for the minimal inception of blood feeding. By contrast, slow responders showed a clear rise in CAA levels, peaking at weeks 4–8 before declining, but still not to background levels. However, the few worms recovered by perfusion at 20 weeks were small and showed no evidence of blood feeding or reproductive activity. An in vitro assay was developed to test the impact of rhesus plasma on the growth and development of 3-day-old schistosomula. It must be emphasised that this was not an ADCC assay of newly transformed schistosomula, and the readout after culture was the measurement of larval ATP level as an index of viability. Plasma from fast responders caused a detectable reduction in viability by week 8, but not until week 16 with slow responder plasma. A further decrease was detected at week 1 post challenge, declining to a maximum loss at week 4. These data provided clear evidence for a memory response elicited by the bolus of challenge parasites. We conclude from the post-challenge CAA data that although adult worm products initiate immune responses that bring about self-cure, their targets must also be present and accessible to antibodies in developing worms, thus preventing worm establishment.

What are the targets of the immune response in self-curing macaques? Characterisation of responses after self-cure from *S. japonicum* implicated the schistosome esophagus as a target for macaque antibodies [76]. Investigation of the morphology and secretions of the anterior and posterior esophageal glands (using RNA-Seq, in situ hybridization, and immunocytochemistry) identified >40 gland products comprising numerous MEGs, lysosomal hydrolases, and some potentially cytotoxic products involved in the initial processing of blood [80]. Evidence gathered by electron microscopy and immunocytochemistry indicated that several products served as in vivo targets for antibodies. With this in mind, we designed small 15-mer peptide arrays covering the amino acid sequences from 32 esophageal gland proteins. We used these to screen the reactivity of 22-week sera from self-curing rhesus macaques versus serum from permissive mice and rabbit host with a chronic infection. [80]. Immunodominant regions were evident across species, notably MEGs 4.1, 4.2, 11, and 12, aspartyl protease, and a Tetraspanin 1 loop, while responses to MEGs 8.1C and 8.2C were largely confined to macaques. As proof of principle, three synthetic genes encoding key targets were designed, and one was expressed as a recombinant protein. When used to vaccinate rabbits, it elicited higher antibody titres to the majority of reactive regions than those elicited after prolonged infection. This clearly demonstrated the feasibility of simultaneously priming an animal against the reactive regions of multiple target proteins (see Section 5 below).

We repeated this type of array analysis using high responder and low responder pools of 18-week sera [75] from rhesus macaques self-curing from *S. mansoni* infection [81]. The array design comprised the 55 tegument and alimentary tract proteins described by Farias et al. [33]. Overall, we identified 41 target epitopes that were almost identical in the high and low responder groups, but the intensity of response was twice as strong in high responders. Moreover, apart from Sm25, tegument proteins elicited much weaker responses than those originating in the alimentary tract. MEGs 41., 4.2, 8.1, 8.2, and 12 were prominent among esophageal gland proteins, whilst asparaginyl endopeptidase and cathepsin B.1.2 were reactive gastrodermal products [81]. These observations reinforced the importance of antibody titre as much as the nature of the target epitope/protein in the self-cure process. A systems biology approach has not yet been applied to the self-cure process in rhesus macaques, but it could highlight differences in host immune responses and metabolism associated with fast and slow self-curing animals. This is essential information for effective vaccine development.

## 5. The Message for Vaccine Development

The animal models reviewed here are concrete examples of the way in which an acquired immune response can ultimately eliminate intra-mammalian schistosomes. The thesis is that in vivo events provide the best prospectus for what a vaccine must achieve to succeed. My digest of the knowledge accumulated over 60 years is summarised below.

### 5.1. Schistosomes Are Hard to Kill In Vivo

- There is minimal evidence for killing of invading larvae in the skin in any model system, attractive as that might be. If slow development of immunity in humans is based on IgE responses, then it is emphatically not a route to a vaccine.
- After arming of the mouse (and rat) lungs by CD4+ T cells caused by a single dose of the RA vaccine, protection depends on blocked migration with some larval deflection into the alveoli. It is possible that the antibody-mediated protection displayed by IFNgR KO mice [33] works in the same way as the early elimination of challenge parasites observed in fast responder macaques [79]. Certainly, the larvae are not killed in the RA vaccinated mouse, but their progress is blocked.
- Antibody-mediated killing of adult worms by self-curing rhesus macaques is a protracted process of several weeks, involving immune pressure on multiple targets and differing specificities between individual animals due to MHC restriction of antigen presentation.
- Only in the rat self-cure process is there rapid and acute elimination of pre-adults. Soluble host-derived mediators (NO, histamine, serotonin, proteases) are the most likely agents, but the requirement for IgE and mast cells is not amenable to vaccine technology. However, the extended lifespan of worms in iNOS rats suggests a weak point that effector responses generating NO might usefully exploit.

### 5.2. What Are the Targets?

- The primary criterion is that the target(s) must be accessible to immune effectors in living parasites. Among current/recent human vaccine candidates, glutathione-S-transferases and fatty acid-binding protein Sm14 are located in the cytosol. The tetraspanin TSP-2 loops are likely exposed and accessible on the tegument surface. Smp80 calpain, while it lacks a signal peptide, is located at the tegument surface, and results from baboon vaccine experiments indicate it is accessible to antibodies [82].
- In recent years, transcriptomic and proteomic studies have identified a significant number of tegument surface constituents and components of esophageal gland and gastrodermal secretions that are largely untested in vaccine experiments (as many as 50 or 60). In parenthesis, there have been some very uncritical proteomic analyses of worm fractions and these issues have been dealt with in recent reviews [74,83].
- One unusual feature of the exposed proteins, revealed by peptide array analysis, is their often very low immunogenicity. Indeed, a bioinformatic analysis of MEG and VAL protein evolution across schistosome species suggests that they have been selected for immunological silence [84].
- Protection mediated by multiple targets seems more probable than a single magic bullet antigen. The feasibility of multi-epitope constructs has been demonstrated and these are now at the design/implementation stage for protection experiments.

### 5.3. What Does a Vaccine Need to Achieve?

- Negotiating the pulmonary vascular bed presents an obstacle in mice and rats that might be exploitable, and immunity conferred by arming of the lungs with memory/effector T cells appears to be reasonably persistent. Use of modified BCG as a vehicle, incorporating a multi-epitope construct, provides a potential way of achieving this by vaccination. Immunisation of mice with the rBCG-LTAK63 vaccine was recently shown to induce a persistent increase in memory and effector T cell numbers in lymph nodes and the lungs for at least 6 months after administration, which correlated with increased protection against *Mycobacterium tuberculosis* [85].
- Self-cure in the rhesus macaque offers the most as a paradigm for a human vaccine, but a strategy of eliciting persistent high titres and a rapid recall response from memory cells is paramount. The development of adjuvants that can accomplish this is an active field with the introduction of products like the synthetic glucopyranosyl lipid A (GLA) agonist of toll-like receptor-4 (TLR-4) [86]. This adjuvant has been extensively used in numerous studies with the Smp80 vaccine (e.g., [87]). Other options being developed include a nanoparticulate comprising bacterial outer membrane vesicles of *Neisseria lactamica* conjugated with biotin and decorated with expressed recombinant schistosome protein in fusion with biotin-binding rhizavidin [88].
- We do not knowwhether any of these formulations can maintain antibody titres against schistosome targets at levels high enough to stress worms to the point that they expire and simultaneously establish a memory population of B cells that can respond sufficiently rapidly to incoming larvae to arrest their migration and development. Experiments are underway in mice and rhesus macaques to test whether these criteria can be fulfilled using multi-epitope constructs. Persistence of protection for a minimum of six months seems to be a reasonable goal.
- A recent rhesus macaque experiment [79] indicated that a recall response in fast responder animals could be detected by one week after challenge. This was conducted with 700 cercariae, representing a biomass of ~25 μg protein. The developing larvae begin blood feeding and releasing esophageal and gastrodermal secretions in μg amounts from around day 8 [83], clearly sufficient to trigger a memory response. In the real world of a community living in an endemic region, most encounters will be with very small numbers of infective larvae on a sporadic basis. Can such larvae activate a sufficient memory response with their ng quantities of secretions or will they simply “slip in under the radar”?

The recent development of a controlled human infection model (CHIM; [89]) should provide the means to answer the questions posed above as a precursor to complex and expensive Phase II/III field trials of human schistosome vaccines. The model involves exposing human volunteers to cercariae of one sex, thus minimising pathology. Highly sensitive assays based on CAA permit the detection of very small numbers of worms resulting from exposure to 10 to 30 male cercariae. The administration of vaccines/placebos to test and control human volunteers accompanied by monitoring of appropriate immune parameters such as specific antibody titre, followed by challenge with cercaria of one sex and monitoring of CAA levels, will hopefully facilitate rapid progress towards a feasible vaccine.

## Data Availability

Not applicable.
